# Weight Loss After Bariatric Surgery Significantly Improves Carotid and Cardiac Function in Apparently Healthy People with Morbid Obesity

**DOI:** 10.1007/s11695-020-04686-y

**Published:** 2020-06-03

**Authors:** Alessandro Giudici, Carlo Palombo, Michaela Kozakova, Carmela Morizzo, Lorenzo Losso, Monica Nannipieri, Rossana Berta, Alun D. Hughes, J. Kennedy Cruickshank, Ashraf W. Khir

**Affiliations:** 1 Department of Mechanical and Aerospace Engineering, Brunel University London, Kingston Lane, Uxbridge, Middlesex UB8 3PH UK; 2 Department of Surgical, Medical, Molecular Pathology and Critical Area Medicine, University of Pisa, Pisa, Tuscany Italy; 3 Department of Clinical and Experimental Medicine, University of Pisa, Pisa, Tuscany Italy; 4 Bariatric Surgery Division, Azienda Ospedaliero Universitaria Pisana, Pisa, Tuscany Italy; 5 Department of Population Science & Experimental Medicine, Institute of Cardiovascular Science, University College London, London, Middlesex UK; 6 School of Life-Course/Nutritional Sciences, King’s College, St. Thomas’ & Guy’s Hospitals, London, Middlesex UK

**Keywords:** Bariatric surgery, Obesity, Carotid artery, LV function, Carotid local PWV, lnDU-loop

## Abstract

**Purpose:**

Obesity clearly increases cardiovascular risk, often inducing high blood pressure (BP), impaired left ventricular (LV) function, and increased arterial stiffness. Intensive weight loss and bariatric surgery induce improvement in hypertension and diabetes for morbid obesity. Carotid artery haemodynamics is a powerful prognostic indicator for stroke and cognitive decline independent of BP. The aim of this study was to evaluate the impact of a 3-stage bariatric strategy of diet, bariatric surgery, and consequent weight loss on carotid haemodynamics and cardiac diastolic function.

**Material and Methods:**

This prospective study included 26 patients (45 ± 10 years, 4 men) with severe obesity undergoing bariatric surgery without comorbidities (hypertension, diabetes, etc.). Anthropometry, BP, Doppler echocardiography, and common carotid haemodynamics by ultrasound were measured at three times: (1) baseline, (2) after 1-month diet (post-diet), and (3) 8 months after surgery (post-surgery). The lnDU-loop method was used to estimate local carotid pulse wave velocity (_*nc*_PWV).

**Results:**

Baseline BMI was 47.9 ± 7.1 kg/m^2^ and reduced by 5% and 30% post-diet and post-surgery, respectively. BP decreased only post-diet, without pulse pressure change. However, _*nc*_PWV, 6.27 ± 1.35 m/s at baseline, was significantly reduced by 10% and 23% post-diet and post-surgery, respectively, also adjusted for BP changes. The E/A ratio rose from 0.95 ± 0.20 to 1.27 ± 0.31 (*p* < 0.005), without change in LV geometry or mass, while heart rate and cardiac output fell substantially.

**Conclusion:**

Weight loss following diet and bariatric surgery is associated with reduced carotid arterial stiffness and improved LV diastolic function. Diet and bariatric surgery are effective treatments for morbid obesity with its concomitant adverse cardiovascular effects.

## Introduction

Increased body weight, a global epidemic, drives cardiovascular risk, such as increased blood pressure (BP), dyslipidaemia, and intolerance to glucose [1, 2], and is associated with increased incidence of type 2 diabetes, hypertension, and cardiovascular diseases. Observational clinical studies and trials show a relationship between weight loss and decrease in BP, cholesterol concentration, and improved glycaemic control [3, 4], as well as increased compliance of main conduit elastic arteries [5].

Arterial stiffness, estimated as aortic pulse wave velocity, predicts cardiovascular events and mortality independently of other standard risk factors [6], including hypertension and diabetes [7], and obesity is associated with increased arterial stiffness [8]. Balkestein and colleagues reported a 10% increase of carotid compliance (the inverse of stiffness) associated with a 15% decrease in body mass index (BMI) [5]. Similar findings were reported for carotid-femoral (cfPWV) and brachial-ankle PWV (baPWV) [9]. In most of these studies, the decrease in BMI was achieved by diet only. Moreover, since arterial stiffness is pressure-dependent in many studies, it is not clear to what extent the reduction in stiffness observed after weight loss merely reflects a parallel decrement in BP.

Bariatric surgery is the most effective treatment for morbid obesity in terms of weight loss, disappearance or improvement of diabetes, hypertension, or dyslipidaemia; the number of procedures per year steadily increases [10]. To our knowledge, no study has examined the effect of bariatric surgery on local arterial stiffness and cardiovascular haemodynamics. Here, we studied the effect on carotid arterial mechanics and ventricular function firstly of 1 month’s weight loss after diet, then secondly approximately 8 months after bariatric surgery in otherwise uncomplicated subjects with morbid obesity. We hypothesised that carotid distensibility and PWV would improve following diet and following surgery, independent of any BP change, as would cardiac indices.

## Methods

### Study Sample and Acquisition Protocol

This prospective study in the University Hospital of Pisa (Tuscany, Italy) included 26 patients (45 ± 10 years, 5 men) referred for bariatric surgery with third-degree obesity (BMI ≥ 40 kg/m^2^). The final study population comprised 32 recruited patients; however, 2 patients dropped out of the study after the diet period and 4 were excluded for inadequate carotid distension waveforms.

All were free of hypertension (systolic BP (SBP) ≥ 140 mmHg and/or diastolic (DBP) ≥ 90 mmHg), diabetes mellitus, atrial fibrillation, heart failure, or previous ischaemic cardiac and cerebral events. Patients with any significant systemic disease were excluded. Five patients had impaired glucose tolerance (IGT) at an oral GT test before the study. All subjects were evaluated at (1) baseline, (2) after 1 month’s diet and before the surgical procedure (post-diet), and (3) 5–11 months (average 8 months) after bariatric surgery (post-surgery). Anthropometry, BP measurements, and ultrasound (US)/echocardiographic examinations were performed on each occasion.

### Anthropometric and BP Measurements

Body weight and height were measured, and BMI and body surface area (BSA) were calculated [11]. Brachial BP was measured by a digital electronic manometer (Omron, model 705cp, Kyoto, Japan), with a suitable adult-size cuff according to arm circumference, after sitting for > 10 min.

### Cardiac and Vascular Ultrasound Studies

All echocardiographic and carotid ultrasound examinations were performed by a single trained sonographer (C.M.), with patients lying supine in a quiet room for > 10 min before the exams. Data were collected using a Hitachi Aloka Alpha10 Prosound system.

### Echocardiographic Examination

Stroke volume (SV) was calculated as the product of aortic valve cross-sectional area and transaortic flow-velocity time integral. Cardiac output (CO) was calculated as the product of SV and heart rate (HR). The total peripheral resistance (TPR) was estimated as the ratio between mean BP (MBP) and CO. Left ventricular (LV) inner diameter and wall thickness were measured in end-diastole from M-mode images [12], and LV mass index was calculated as the ratio of LV mass to height^2.7^ [13]. Relative wall thickness (RWT) was calculated as a ratio between wall thickness and inner diameter.

Indices of LV diastolic function were obtained by mitral inflow measurements including early peak filling (E velocity), late peak atrial filling (A velocity), and E/A ratio [14].

Systolic and diastolic mitral longitudinal velocities at both the septal and lateral sides were measured by colour-guided pulsed-wave tissue Doppler in the apical four-chamber view. The sample volume was placed at the junction of the LV wall with the mitral annulus, and the cursor was aligned so that the angle of incidence between the Doppler beam and the longitudinal motion of the LV was as close as possible to 0°. From spectral traces, peak longitudinal velocities during systole (s′), during early diastolic filling (e′), and during late diastolic filling (a′) were measured over five consecutive cardiac cycles. Reported values represent the average of septal and lateral sides. The intra-individual variability of tissue Doppler measurements in our laboratory is 4.7 ± 3.5%, 5.8 ± 4.3%, and 5.2 ± 4.0% for s′, e′, and a′ velocities, respectively.

### Carotid Waveform Acquisition

Flow velocity and distension waveforms were acquired at the level of the right common carotid artery (CCA) using a 10.0-MHz linear array probe with radiofrequency data output at the frequency of 1 kHz, as previously reported [15]. In the longitudinal right CCA view, a single scan line was aligned perpendicularly to the vessel walls, approximately ≈ 1.5 cm proximal to the carotid bulb; the cursors were placed by the operator at the anterior and posterior carotid walls to enable wall tracking. After 20 s of acquisition, all distension curves and flow profiles were displayed, and individual beats with noisy or unrepresentative waveforms were rejected by the operator before ensemble averaging.

### Determination of Non-invasive Local Carotid Pulse Wave Velocity and Arterial Distensibility

The local carotid pulse wave velocity (_*nc*_PWV (*c*)) was determined using the lnDU-loop method (Eq. 1) as previously described in detail [16]:

1 $$ c=\pm \frac{1}{2}\frac{\mathrm{d}{\mathrm{U}}_{\pm }}{d\left(\ln {\mathrm{D}}_{\pm}\right)} $$

where subscripts + and – indicate forward and backward directions of wave travel, respectively, i.e. waves travelling from the heart towards the peripheral circulation and reflected waves travelling back from the periphery to the heart. Also, dU and dlnD represent the instantaneous changes in the blood velocity U and the natural logarithm of the diameter. The relationship between U and lnD (Fig. 2) is linear in early systole whose slope was used for the calculation of *c* (Eq. 1).

The relationship between _*nc*_PWV and local arterial distensibility (*D*_s_) is described by the Bramwell-Hill equation:

2 $$ {D}_{\mathrm{s}}=\frac{1}{\rho {c}^2} $$

where *ρ* is the blood density (assumed 1060 kg/m^3^) and *c* is calculated using Eq. 1.

#### The Separation of D and U into Forward and Backward Components and Wave Intensity I

WIA allows separation of wave intensities travelling to the forward from the backward directions [16]. Wave intensity (dI) is given by the product of dD and dU. Therefore, forward and backward intensities are given by:

3 $$ \mathrm{d}{\mathrm{I}}_{\pm }=\mathrm{d}{\mathrm{D}}_{\pm}\mathrm{d}{\mathrm{U}}_{\pm } $$

**Fig. 1:**
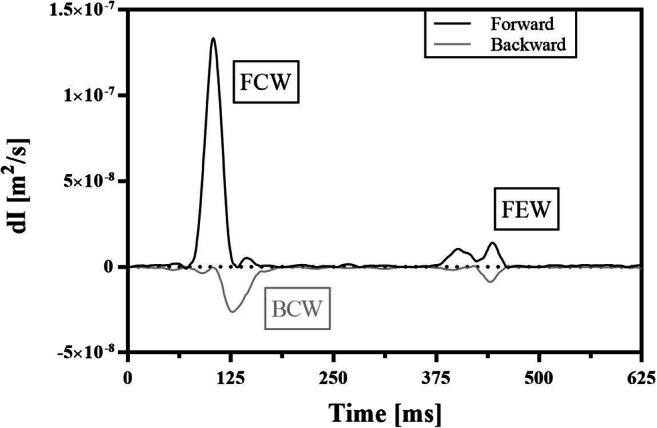
Example of wave intensity for one of the subjects (not showing late diastole when wave intensity is minimal). The wave intensity (dI) pattern is characterised by three main peaks: forward compression (FCW) and forward expansion waves (FEW), and a backward compression wave (BCW). See “Methods” for details

By definition, dI_−_ is always negative, while dI_+_ is always positive. As in Fig. 1, the forward wave intensity is characterised by two main waves; the first is a large forward compression wave (FCW) related to the ejection phase of the heart. The second is later, a forward expansion wave (FEW) prior to aortic valve closure and contemporaneous with the end of ejection. dI_−_ is mainly characterised by a small peak from a backward compression wave (BCW) resulting from reflection of the FCW from downstream arterial sites during early systole. These three intensity peaks have been characterised in terms of underlying area and arrival time. The reflection index, the portion of the forward travelling wave that is reflected back from the periphery to the heart, was calculated as the ratio between BCW and FCW areas.

### Statistical Analysis

IBM SPSS statistics (IBM Corporation, Armonk, NY, USA) was used for the statistical analysis. Data presented a normal distribution and are reported as mean ± SD. Changes in anthropometric and BP measurements and cardiac parameters were evaluated using repeated measures ANOVA. If a significant change was found, the three treatment steps were compared using 2-tailed paired samples *t* tests. *p* ≤ 0.05 was considered statistically significant. A similar analysis was performed also on the wave intensity parameters FCW area, BCW area, FEW area, and relative timing. Repeated measures ANCOVA was used to evaluate changes in _*nc*_PWV adjusting for DBP changes during treatment. Analysis of variance or multiple regressions were then used as appropriate.

## Results

### Treatment Outcomes

The average basal weight, BMI, and BSA of the study participants were 129.9 ± 27.0 kg, 47.9 ± 7.1 kg/m^2^, and 2.29 ± 0.28 m^2^, respectively. The 1-month diet period produced a ≈ 5% reduction in body weight and BMI, and a ≈ 2% reduction in BSA. After the 8-month follow-up post-bariatric surgery, the weight and BMI dropped by 30 ± 8% of the basal value, while the decrease in BSA was 14 ± 4% (Table 1).

**Table 1:** Physical and haemodynamics features of subjects included in the study. Participant characteristics: baseline, post-diet, and post-surgery values. Values are mean ± SD. Pressure was recorded at the brachial artery. *SBP*, systolic BP; *DBP*, diastolic BP. *p < 0.05, ^†^*p* < 0.01 with respect to basal value; ^‡^*p* < 0.05, ^§^*p* < 0.01 with respect to diet

	Baseline	Post-diet	Post-surgery	rm-ANOVA
Body weight (kg)	129.9 ± 27.0	123 ± 24.8^†^	90.5 ± 21.3^†,§^	*p* < 0.005
BMI (kg/m^2^)	47.9 ± 7.1	45.5 ± 6.7^†^	33.4 ± 6.9^†,§^	*p* < 0.005
BSA (m^2^)	2.29 ± 0.28	2.24 ± 0.26^†^	1.96 ± 0.24^†,§^	*p* < 0.005
SBP (mmHg)	122.2 ± 13.3	116.3 ± 12.4^†^	115.7 ± 16.8*	*p* < 0.05
DBP (mmHg)	81.5 ± 7.5	78.1 ± 6.2^†^	77.0 ± 9.0*	*p* < 0.05
PP (mmHg)	40.7 ± 10.0	38.2 ± 9.4	38.7 ± 10.4	*p* = 0.35
Mean BP (mmHg)	91.7 ± 8.2	87.6 ± 7.1^†^	86.7 ± 10.5*	*p* < 0.05

There was a 5.5% decrease in SBP and DBP over the whole length of the study. However, BP decreased mainly during the initial 1-month diet period, and there was little further change at the 8-month follow-up after bariatric surgery.

### Cardiac Structure and Function

Baseline, post-diet, and post-surgery cardiac indices are reported in Table 2. HR was 76 ± 10 bpm at baseline and decreased significantly post-diet (10 ± 11%) and post-surgery (20 ± 10%). Basal SV was 92 ± 18 mL and did not change significantly post-diet or post-surgery, whereas CO decreased post-diet (17 ± 13%) and post-surgery (21 ± 17%), due to the decline in HR. Baseline EF was 68% and did not change with the treatment. TPR increased significantly after diet and surgery (*p* < 0.001 for both). LV mass, LVMI, and RWT did not change post-diet or post-surgery. Early diastolic e′ and late diastolic a′ velocities of the mitral annulus significantly increased and decreased, respectively, post-surgery. Mitral E and A velocities showed similar trends, so that the E/A ratio significantly increased (36 ± 30%) during the treatment, while the E/e′ ratio did not change post-diet and post-surgery. Considering the three observational time points, i.e. baseline, post-diet, and post-surgery, e′ velocity and E/A ratio were inversely related to _*nc*_PWV (*r* = − 0.37, *p* < 0.001 and − 0.32, *p* < 0.01, respectively). Moreover, E and E/A ratio negatively correlated with HR (*r* = − 0.29, *p* < 0.01 and − 0.44, *p* < 0.001, respectively).

**Table 2:** Cardiac indices at baseline, post-diet, and post-surgery. Values are presented as mean ± SD. *SV*, stroke volume; *CO*, cardiac output; *EF*, ejection fraction; *LV mass*, left ventricular mass; *LVMI*, left ventricular mass index; *TDI*, tissue Doppler imaging. s′, e′, and a′ velocities are systolic, early diastolic, and late diastolic transmitral velocity, respectively. *p < 0.05, ^†^*p* < 0.01 with respect to basal value; ^‡^*p* < 0.05, ^§^*p* < 0.01 with respect to diet

	Baseline	Post-diet	Post-surgery	rm-ANOVA
HR (bpm)	76 ± 10	69 ± 10^†^	61 ± 10^†,§^	*p* < 0.005
SV (mL)	92 ± 18	84 ± 17	89 ± 16	*p* = 0.09
CO (L/min)	7.0 ± 1.5	5.8 ± 1.2^†^	5.4 ± 1.2^†,‡^	*p* < 0.005
EF [-]	68 ± 5	65 ± 4	67 ± 5	*p* = 0.093
TPR (mmHg/L/min)	13.6 ± 2.9	15.9 ± 3.5^†^	16.8 ± 3.3^†^	*p* < 0.005
LV mass (g)	204 ± 53	206 ± 51	202 ± 41	*p* = 0.89
LVMI (g/m^2^)	53 ± 12	54 ± 10	53 ± 11	*p* = 0.94
RWT [-]	0.37 ± 0.05	0.37 ± 0.04	0.36 ± 0.05	*p* = 0.69
TDI s′ velocity (cm/s)	9.8 ± 1.3	9.4 ± 1.4	9.4 ± 1.4	*p* = 0.19
TDI e′ velocity (cm/s)	12.7 ± 2.8	12.9 ± 2.4	14.3 ± 3.1^†,§^	*p* < 0.005
TDI a′ velocity (cm/s)	13.2 ± 2.3	12.6 ± 1.8	11.5 ± 1.9^†,§^	*p* < 0.005
Mitral E velocity (cm/s)	68.9 ± 17.2	75.3 ± 19.4	84.0 ± 18.6	*p* < 0.005
Mitral A velocity (cm/s)	74.7 ± 19.9	74.3 ± 21.8	69.3 ± 19.5*^,‡^	*p* < 0.05
TDI e′/a′ ratio	0.98 ± 0.25	1.04 ± 0.22	1.28 ± 0.36^†,§^	*p* < 0.005
E/A ratio	0.95 ± 0.20	1.05 ± 0.22*	1.27 ± 0.31^†,§^	*p* < 0.005
E/e′ ratio	5.78 ± 2.15	6.08 ± 2.13	6.14 ± 1.91	*p* = 0.20

### Local PWV

**Fig. 2:**
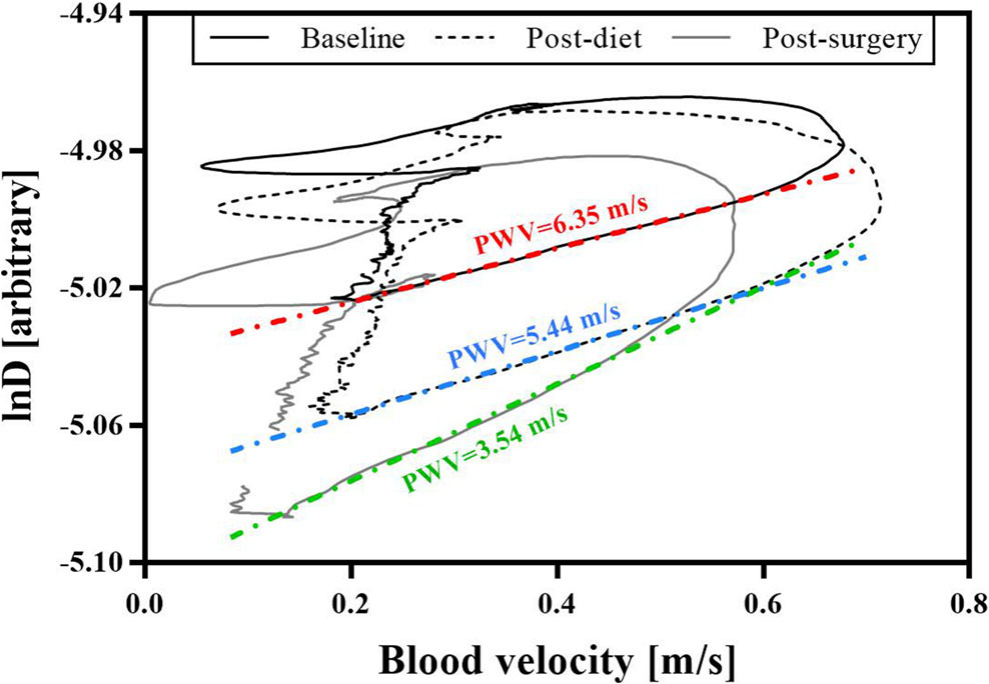
The carotid lnDU-loop measured at baseline, post-diet, and post-surgery. The linear regression of the early systolic portion of the loop is shown for each lnD-U relationship. The slope of the early systolic portion of the loop slightly increased after the diet and increased more markedly after the surgical procedure. _*nc*_PWV was 6.35 m/s, reduced to 5.44 after the diet and to 3.54 m/s after the surgery. Carotid pulse diameter, expressed as the difference between maximum and minimum diameters, increased significantly from basal to post-diet and post-surgery acquisition. On the other hand, the pulse velocity (U_max_-U_min_) remained approximately constant

Baseline, post-diet, and post-surgery haemodynamic indices are reported in Table 3. Figure 2 shows the lnDU-loops, from which _*nc*_PWV is calculated, at baseline, post-diet, and post-surgery in a typical subject in the study. On average at baseline, _*nc*_PWV was 6.27 ± 1.35 m/s. On average, diet produced a significant decrease in _*nc*_PWV by 10 ± 14%. The difference was still significant after adjusting for BP effects, but decreased to 9%.

**Table 3:** Haemodynamic indices at baseline, post-diet, and post-surgery. *D*_*max*_, maximum carotid diameter; *D*_*min*_, minimum carotid diameter; *∆D*, D_max_-D_min_; *U*_*max*_, maximum carotid velocity; *U*_*min*_, minimum carotid velocity; _*nc*_*PWV*, non-invasive carotid pulse wave velocity; *D*_*s*_, carotid distensibility. *p < 0.05, ^†^*p* < 0.01 with respect to basal value; ^‡^*p* < 0.05, ^§^*p* < 0.01 with respect to diet

	Baseline	Post-diet	Post-surgery	rm-ANOVA
Carotid flow (L/min)	0.60 ± 0.20	0.60 ± 0.17	0.55 ± 0.18	*p* = 0.46
D_max_ (mm)	8.23 ± 1.22	8.11 ± 1.11	7.95 ± 1.18*	*p* < 0.05
D_min_ (mm)	7.76 ± 1.18	7.63 ± 1.06	7.41 ± 1.10^†^	*p* < 0.05
∆D (mm)	0.47 ± 0.11	0.48 ± 0.13	0.54 ± 0.16^†,§^	*p* < 0.01
∆D/D_min_ %	6.08 ± 1.41	6.35 ± 1.69	7.30 ± 1.93^†,§^	*p* < 0.005
U_max_ (m/s)	0.53 ± 0.10	0.55 ± 0.13	0.51 ± 0.09	*p* = 0.50
U_min_ (m/s)	0.06 ± 0.06	0.07 ± 0.07	0.07 ± 0.07	*p* = 0.64
_*nc*_PWV (m/s)	6.27 ± 1.35	5.52 ± 0.93^†^	4.74 ± 1.09^†,§^	*p* < 0.005
*D*_s_ (MPa^−1^)	27.5 ± 11.2	33.6 ± 10.9^†^	50.0 ± 27.0^†,§^	*p* < 0.005

Eight months after the bariatric surgery, _*nc*_PWV was 4.74 ± 1.09 m/s, 23 ± 15% and 14 ± 15% less than at the start and after the 1-month diet period, respectively (22% and 14% after BP adjustment). The difference was statistically significant with respect to both the basal and post-diet measurements.

The relative carotid distension ∆D/D_min_% significantly increased by 20% post-surgery (6.08 ± 1.41 vs. 7.30 ± 1.93) and negatively correlated with _*nc*_PWV (*r* = − 0.57, *p* < 0.001).

### Wave Intensity

**Fig. 3:**
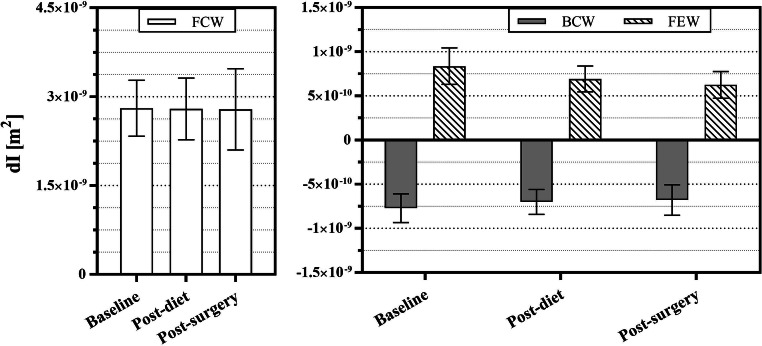
Average forward compression wave (FCW) (left), backward compression wave (BCW), and forward expansion wave (FEW) (right) area at baseline, post-diet, and post-surgery. Results are mean ± 95% confidence interval. BCW and FEW are shown separately (right panel) for clarity

Wave intensity did not show statistically significant changes between different occasions (Fig. 3). FCW was very similar across the three time points. On the other hand, BCW fell slightly from 7.71 ± 1.60·10^−10^ m^2^ at baseline to 6.34 ± 1.90·10^−10^ at 8 months, the end of the study. The trend for the FEW was similar: 8.37 ± 2.02·10^−10^ basally and 6.25 ± 1.49·10^−10^ at 8 months. Consequently, both the reflection index BCW/FCW and the ratio FEW/FCW decreased after the surgery, but these differences were not statistically significant.

The arrival time of the waves was significantly affected by the surgical procedure. The time lag between FCW and BCW was higher in the post-surgery recording than at baseline. The baseline time lag between FCW and BCW was 33.0 ± 10.7 ms, which increased to 40.8 ± 12.2 (*p* < 0.05) and 43.5 ± 14.4 (*p* < 0.005) after 1 month of diet and 8 months after bariatric surgery, respectively.

## Discussion

Arterial stiffness and body weight are generally positively correlated, and weight loss has been associated with decrease in cfPWV and baPWV [17]. However, to the authors’ knowledge, no studies have examined changes in local PWV (_*nc*_PWV) after bariatric surgery. Here, we evaluated the effect of extensive weight loss on cardiac haemodynamics and cardiac structure and function in 26 patients with third-degree obesity carefully selected according to the absence of comorbidities, particularly hypertension and diabetes, which affect cardiovascular structure independently of obesity. We compared data between baseline, post-diet, and post–bariatric surgery.

As much as 40-kg weight loss or 11.7 kg/m^2^ BMI, almost 30% of basal BMI here, was associated with improvements in cardiac performance during systole and diastole. The changes in HR we observed following reduction in BMI are similar to those reported in overweight young adults [17]. Stroke volume [18], measured by Doppler echocardiography, did not decrease significantly, so the significant decrease in CO both post-diet and post-surgery reflects the changes in HR. Contrary to expectation, the lack of change in LV mass and geometry over the study may be explained by the relatively short follow-up period. Previous studies demonstrating LV mass reduction post-surgery had a longer follow-up period [19].

The ≈ 36% increase in E/A ratio is a beneficial effect of weight loss on E velocity (early diastolic transmitral velocity) reported after bariatric surgery [20] but also in the general population, where a 1-unit decrease in BMI during 1-year follow-up led to an increase in early diastolic myocardial velocity (e′ velocity) of 0.11 cm/s [21]. The mechanism underlying this improvement is not clear. Although it has been suggested that an increase in e′ velocity follows the reduction of LV mass [22], in our study, this occurred without observing such reduction. The improvement of LV diastolic function after bariatric surgery, assessed both as transmitral flow velocity pattern and as tissue myocardial velocities, happened in a relatively short time (months), suggesting that it could be mainly depending on functional mechanisms, particularly changes in heart rate. An increase of the transmitral A peak velocity with increasing heart rate was demonstrated by Harrison et al. [23]. Furthermore, the inverse relationship between carotid stiffness and e′ velocity and E/A ratio suggests that arterial stiffness contributes to LV diastolic dysfunction [24] and indicates that reversing local stiffening as judged by _*nc*_PWV improves cardiac performance. Indeed, adjusting for _*nc*_PWV reduced by 100% and 50% the e′ changes obtained after diet and surgery, respectively.

In line with our hypothesis, the ~ 25% reduced _*nc*_PWV at ~ 8 months post-surgery was due to increased relative distension of the carotid wall. Borlotti et al. [25] reported values of _*nc*_PWV in the carotid artery just above 4 m/s using the lnDU-loop method in a healthy 40-year-old sample. Di Lascio et al. [26] found an average _*nc*_PWV of 5.64 ± 1.7 m/s in 50.5 ± 20-year-olds using the foot-to-foot method with accelerometric sensors positioned on the carotid. We used the PD^2^-loop method [27] for pressure and distension waveforms acquired simultaneously on the left and right common carotid arteries giving _*nc*_PWV values of 4.58 ± 0.63 m/s in healthy 40–50-year-olds (unpublished data). Therefore, our results indicate that increased carotid stiffness is a likely consequence of obesity because it does decline, and into the healthy range, after this marked weight loss, in agreement with other groups [5], but here shown to be independent of the BP change. As further support for our hypothesis, only three patients did not show a decrease in _*nc*_PWV between the basal and the post-surgery measurements, and 2 of these already had basal PWVs within the normal range.

Recently, Streese et al. [28] examined the changes in the Cardio-Ankle Vascular Index (CAVI) and baPWV 6 weeks and 4 years after bariatric surgery. Neither of the measures changed significantly, in apparent disagreement with our finding. Similarly, Galkine et al. [29] studied changes in CAVI after bariatric surgery in patients with at least one comorbidity, reporting an increase in CAVI at 1 year and no difference from baseline at a 4-year follow-up. Explanations for this disparity are that CAVI and baPWV are *global* indices of arterial stiffness, including long muscular arterial pathways, while central (aortic) PWV and our lnDU-loop method provide central and here local measures of PWV. Given the difference in wall composition and stiffness of different sites along the arterial bed, local stiffness might be affected differently. In addition, the majority of participants included in their studies were hypertensive, while ours were not.

Arterial stiffness (i.e. PWV) is intrinsically pressure-dependent [30]. In our study, brachial S/D BP decreased by 6/4 mmHg, respectively, during the 1-month diet with a 2.4-kg/m^2^ BMI decrease. Cooper et al. [17] reported a reduction in S/D BP following 1-year lifestyle intervention of diet and physical activity, only about half observed here. Belkestein et al. [5] reported a decrease in MBP comparable with ours, but with twice the change in BMI following a 10-week energy-restricted diet and exercise programme. However, the degree of obesity in their studies was much less than that in ours (baseline BMI = 32.9 and 32.3 kg/m^2^), and the relationship between change in BMI and change in MBP may not be linear. Although the change in BP was significant, its relatively small amplitude was responsible for only 1% of the _*nc*_PWV reduction. Furthermore, here, 8 months post-surgery when BMI had decreased further by 11.1 kg/m^2^, SBP and DBP had not reduced further but there was a further fall in PWV. Other recent results suggest SBP and DBP did significantly decrease 6 months after bariatric surgery [31, 32]. However, the lack of pressure changes with time after the intervention here suggests that much of the average 0.78 m/s reduction in _*nc*_PWV comparing diet with 8-month follow-up is related to changes in arterial wall mechanics.

Interestingly, we did not find any correlation between the magnitude of intra-subject changes in BMI (or weight) and cardiac/vascular parameters (i.e. transmitral flow velocities, mitral a′ and s′ velocities, CO, cPWV, *D*_s_), except for a negative correlation between intra-subject changes in BMI (and weight) and mitral e′ velocity (*r* = − 0.43, *p* < 0.05). Sutton-Tyrell et al. [8] found a higher correlation between aortic stiffness and visceral adiposity than between aortic stiffness and body weight, and postulated the hypothesis that correlation between aortic stiffness and body weight might actually reflect a correlation between body weight and visceral adiposity. Therefore, it is possible that other parameters contributing to determining the body weight may affect changes in cardiac and vascular parameters.

WIA, analysed for the first time in this context here, did not provide as clear a trend as found for _*nc*_PWV. FCW area did not change, while BCW and FEW showed consistent but small decreases. The reduced reflection index may indicate improved transmission of waves to the periphery; however, this difference was not significant (*p* = 0.214) and could be by chance. Our sample size might have been too small to detect clinically significant differences. The difference of arrival time of BCW with respect to FCW suggests, as in the lower _*nc*_PWV, that waves travel slower after weight loss, indicating further potential benefits.

## Limitations

This study is a before-after comparison without a formal control group, but medically treated patients with third-degree obesity never lose the amount of weight found after surgery as here. However, it is theoretically possible that time-dependent changes could occur with this design, but is most unlikely. Our sample size was relatively small but prospective, and no patients had hypertension or type 2 diabetes, a unique feature of our cohort to date allowing assessing cardiovascular changes related to obesity alone. It is possible that diabetes and hypertension might make weight-related arterial stiffening less reversible. Finally, LV diastolic function was assessed simply by E and A peak velocities at transmitral flow velocity pattern, and by the corresponding components at TDI.

## References

[1] Safar ME, Czernichow S, Blacher J (2006). Obesity, arterial stiffness, and cardiovascular risk. J Am Soc Nephrol.

[2] Hubert HB, Feinleib M, McNamara PM, Castelli WP (1983). Obesity as an independent risk factor for cardiovascular disease: a 26-year follow-up of participants in the Framingham Heart Study. Circulation..

[3] Czernichow S, Mennen L, Bertrais S, Preziosi P, Hercberg S, Oppert J (2002). Relationships between changes in weight and changes in cardiovascular risk factors in middle-aged French subjects: effect of dieting. Int J Obes.

[4] Liu RH, Wharton S, Sharma AM, Ardern CI, Kuk JL (2013). Influence of a clinical lifestyle-based weight loss program on the metabolic risk profile of metabolically normal and abnormal obese adults. Obesity..

[5] Balkestein EJ, Van Aggel-Leijssen DP, Van Baak MA, Struijker-Boudier HA, Van Bortel LM (1999). The effect of weight loss with or without exercise training on large artery compliance in healthy obese men. J Hypertens.

[6] Ben-Shlomo Y, Spears M, Boustred C, May M, Anderson SG, Benjamin EJ, Boutouyrie P, Cameron J, Chen C-H, Cruickshank JK, Hwang S-J, Lakatta EG, Laurent S, Maldonado J, Mitchell GF, Najjar SS, Newman AB, Ohishi M, Pannier B, Pereira T, Vasan RS, Shokawa T, Sutton-Tyrell K, Verbeke F, Wang K-L, Webb DJ, Hansen TW, Zoungas S, McEniery CM, Cockcroft JR, Wilkinson IB (2014). Aortic pulse wave velocity improves cardiovascular event prediction. J Am Coll Cardiol.

[7] Cruickshank K, Riste L, Anderson SG, Wright JS, Dunn G, Gosling RG (2002). Aortic pulse-wave velocity and its relationship to mortality in diabetes and glucose intolerance: an integrated index of vascular function?. Circulation..

[8] Sutton-Tyrrell K, Newman A, Simonsick EM, Havlik R, Pahor M, Lakatta E, Spurgeon H, Vaitkevicius P (2001). Aortic stiffness is associated with visceral adiposity in older adults enrolled in the study of health, aging, and body composition. Hypertension..

[9] Petersen KS, Clifton PM, Lister N, Keogh JB (2016). Effect of weight loss induced by energy restriction on measures of arterial compliance. A systematic review. Atherosclerosis..

[10] Angrisani L, Santonicola A, Iovino P, Formisano G, Buchwald H, Scopinaro N (2015). Bariatric surgery worldwide 2013. Obes Surg.

[11] Du Bois D, Du Bois EF (1915). The measurement of the surface area of man. Arch Intern Med.

[12] Marwick TH, Gillebert TC, Aurigemma G, Chirinos J, Derumeaux G, Galderisi M, Gottdiener J, Haluska B, Ofili E, Segers P, Senior R, Tapp RJ, Zamorano JL (2015). Recommendations on the use of echocardiography in adult hypertension: a report from the European Association of Cardiovascular Imaging (EACVI) and the American Society of Echocardiography (ASE). Eur Heart J Cardiovasc Imaging.

[13] De Simone G, Daniels SR, Devereux RB, Meyer RA, Roman MJ, De Divitiis O, Alderman MH (1992). Left ventricular mass and body size in normotensive children and adults: assessment of allometric relations and impact of overweight. J Am Coll Cardiol.

[14] Nagueh SF, Smiseth OA, Appleton CP, et al. Recommendations for the evaluation of left ventricular diastolic function by echocardiography: an update from the American Society of Echocardiography and the European Association of Cardiovascular Imaging. J Am Soc Echocardiogr 2016;29(4):277–314. 10.1016/j.echo.2016.01.011 27037982

[15] Uejima T, Dunstan FD, Arbustini E, et al. E-tracking international collaboration group (ETIC). Age-specific reference values of carotid arterial stiffness estimated by ultrasonic wall tracking. J Hum Hypertens. 2019; 10.1038/s41371-019-0228-5.

[16] Feng J, Khir AW (2010). Determination of wave speed and wave separation in the arteries using diameter and velocity. J Biomech.

[17] Cooper JN, Buchanich JM, Youk A, Brooks MM, Barinas-Mitchell E, Conroy MB, Sutton-Tyrrell K (2012). Reductions in arterial stiffness with weight loss in overweight and obese young adults: potential mechanisms. Atherosclerosis..

[18] van Brussel PM, van den Bogaard B, de Weijer BA, Truijen J, Krediet CTP, Janssen IM, van de Laar A, Kaasjager K, Fliers E, van Lieshout JJ, Serlie MJ, van den Born B-HJ (2017). Blood pressure reduction after gastric bypass surgery is explained by a decrease in cardiac output. J Appl Physiol.

[19] Graziani F, Leone AM, Cialdella P, Basile E, Pennestrì F, Della Bona R, Iaconelli A, Liuzzo G, Biasucci LM’, Cardillo MT, Iaconelli A, Guidone C, Nanni G, Mingrone G, Crea F (2013). Effects of bariatric surgery on cardiac remodeling: clinical and pathophysiologic implications. Int J Cardiol.

[20] Hsuan C, Huang C, Lin L, Lee T, Tai C, Yin W, Tseng W, Hsu K, Wu C (2010). The effect of surgical weight reduction on left ventricular structure and function in severe obesity. Obesity..

[21] Lee SC, Daimon M, Di Tullio MR, Homma S, Hasegawa T, Chiou SH, Nakao T, Hirokawa M, Mizuno Y, Yatomi Y, Yamazaki T, Komuro I (2018). Beneficial effect of body weight control on left ventricular diastolic function in the general population: an analysis of longitudinal data from a health check-up clinic. Eur Heart J Cardiovasc Imaging.

[22] De las Fuentes L, Waggoner AD, Mohammed BS, Stein RI, Miller BV, Foster GD, Wyatt HR, Klein S, Davila-Roman VG (2009). Effect of moderate diet-induced weight loss and weight regain on cardiovascular structure and function. J Am Coll Cardiol.

[23] Harrison MR, Clifton GD, Penneil AT, DeMaria AN (1991). Effect of heart rate on left ventricular diastolic transmitral flow velocity patterns assessed by Doppler echocardiography in normal subjects. Am J Cardiol.

[24] Kim H, Lim W, Seo J, Chung W, Kim S, Kim M, Zo J (2017). Association between arterial stiffness and left ventricular function in relation to gender and age. Medicine..

[25] Borlotti A, Khir AW, Rietzschel ER, De Buyzere ML, Vermeersch S, Segers P (2012). Noninvasive determination of local pulse wave velocity and wave intensity: changes with age and gender in the carotid and femoral arteries of healthy human. J Appl Physiol.

[26] Di Lascio N, Bruno R, Stea F, Bianchini E, Gemignani V, Ghiadoni L, Faita F (2014). Non-invasive assessment of carotid PWV via accelerometric sensors: validation of a new device and comparison with established techniques. Eur J Appl Physiol.

[27] Alastruey J (2011). Numerical assessment of time-domain methods for the estimation of local arterial pulse wave speed. J Biomech.

[28] Streese L, Königstein K, Goricki L, Infanger D, Wölnerhanssen B, Peters T, Schmidt-Trucksäss A, Hanssen H (2019). Short- and long-term effects of bariatric surgery on vascular phenotype. Obes Surg.

[29] Galkine A, Dzenkeviciute V, Sapoka V, Urbanavicius V, Petrulioniene Z, Brimas G, Laucevicius A (2018). Effect of body weight reduction on the arterial stiffness and endothelial function after bariatric surgery in morbidly obese patients: a 4-year clinical study. Acta Endocrinol.

[30] Spronck B, Heusinkveld M, Vanmolkot F, Roodt JO, Hermeling E, Delhaas T, Kroon AA, Reesink KD (2015). Pressure-dependence of arterial stiffness: potential clinical implications. J Hypertens.

[31] Gluszewska A, Gryglewska B, Gąsowski J, Bilo G, Zarzycki B, Dzieża-Grudnik A, Major P, Budzyński A, Faini A, Parati G, Grodzicki T (2019). Reduction of 24-h blood pressure variability in extreme obese patients10 days and 6 months after bariatric surgery depending on pre-existing hypertension. Eur J Intern Med.

[32] Hawkins D, Faler B, Choi Y, Prasad B (2018). Time course of blood pressure decrease after bariatric surgery in normotensive and hypertensive patients. Obes Surg.

